# Precision Oral Health: Potential Applications and Challenges

**DOI:** 10.1055/s-0046-1816688

**Published:** 2026-11-03

**Authors:** Ensaldo-Carrasco E., Chen A., Mun M., Chaturvedi J., Shimpi N.

**Affiliations:** 1 Centro de Investigaciones en Ergonomía, Universidad de Guadalajara, GuadalajaraJalisco; 2 Sir John Walsh Research Institute, Faculty of Dentistry, University of Otago, DunedinNew Zealand; 3 Melbourne Dental School, Faculty of Medicine, Dentistry and Health Sciences, University of MelbourneAustralia; 4 Institute of Psychiatry, Psychology, and Neuroscience, King's College London, LondonUnited Kingdom; 5 Marshfield Clinic Health System, Marshfield, WIUSA

**Keywords:** Precision Medicine, Oral Health, Artificial Intelligence, Patient-centred Care

## Abstract

**Objectives** : This position paper explores the current landscape, potential applications, and key challenges related to precision health in dentistry. In addition, the paper demonstrates how data-driven, patient-centred approaches can be utilized in dentistry to drive continuous quality improvement and inform strategic decision-making.

**Methods** : A comprehensive literature review was undertaken to synthesize ongoing developments in precision oral healthcare. This review encompasses the integration of digital health technologies, emerging standards, and considerations for interoperability in the field of dentistry.

**Results** : While precision health is increasingly influencing oral healthcare, the literature showed that its adoption in dentistry trails behind other medical specialties in adopting such approaches. Key priorities for advancing precision health in dentistry include the integration of patients' genetic information, the application of artificial intelligence, and the use of advanced data analytics. Yet, several significant barriers remain, including a lack of standardized data formats, limited interoperability among health information systems, and the need for strong evidence to support the development and implementation of emerging technologies. To overcome these challenges, it will require coordinated efforts and collaboration among dental providers, healthcare professionals, informaticians, and researchers.

**Conclusion** : Precision oral health holds significant promise for improving patient outcomes through tailored, data-driven interventions. Solutions are needed to address the adoption of technologies, fostering data standards in clinical data, and collaboration across disciplines. Ongoing research and strategic investment in dental informatics are crucial for advancing the integration of precision health principles into routine dental practice.

## 1. Introduction

Patient-centered care is an essential component of healthcare systems in which approaches and disciplines to foster/improve patient-centered care have emerged, particularly from the field of informatics. This has resulted in the emergence and consolidation of medical informatics. As a result, the resolution WHA58.28 issued in 2005 by the World Health Assembly (WHA) urged member states to develop and implement eHealth services and develop the infrastructure for information and communication technologies for health [ 1 ]. In response, researchers from member states have commenced developing the field of precision healthcare, among other areas. Precision healthcare is a cutting-edge approach that leverages the latest technological advancements to promote and maintain optimal health and well-being for the population. By utilizing a comprehetnsive system of continuous health data monitoring, precision healthcare practitioners can detect early warning signs of potential health issues and make proactive interventions to prevent the onset of chronic disease or disability. The personalized nature of precision healthcare ensures that individuals receive tailored interventions uniquely suited to their specific needs, leading to better health outcomes and enhanced quality of life [ 2 3 ]. Precision medicine aims to provide personalized care tailored to each patient's unique needs, resulting in more effective and efficient treatment outcomes through data [ 4 ]. In addition, it employs an individual's genetic profile and artificial intelligence to inform decisions toward disease prevention, diagnosis, and treatment [ 5 ].

When compared to precision medicine, precision health involves a more comprehensive approach to health monitoring that considers an individual's entire lifespan. This approach aims to identify potential health risks and take early action to address them through various interventions such as ongoing health screening, early diagnostic testing, and support for improving one's behavior and adopting a healthier lifestyle. Precision health can help individuals maintain optimal health and well-being throughout their lives by focusing on proactive measures. As many countries underwent a digitalization process, healthcare professionals worldwide became interested in exploring the potential applications of digital technologies in health. Precision dentistry, considered part of precision health, encompasses a multifaceted, data-driven approach to oral and dental health ( Figure 1 ). However, compared to medicine, dentistry has been adopting these new technologies at a slower pace. Dentistry has lagged behind medicine in adopting and seamlessly integrating new technologies, such as precision dentistry and data science [ 6 ], that can potentially improve oral health outcomes. However, with the emergence of precision dentistry and data science, there is an opportunity to enhance the accuracy and effectiveness of dental procedures, leading to better oral health for patients. These data sources include information from genomics, lifestyle factors, environmental conditions, and social media interactions, among others. By gathering and analyzing data from these varied sources, precision health practitioners can gain a comprehensive understanding of an individual's health status and tailor treatments to their unique needs [ 7 ]. Therefore, in this position paper, we aim to provide a comprehensive overview of the applications and challenges of precision prevention in the field of dentistry.

**Figure 1.**
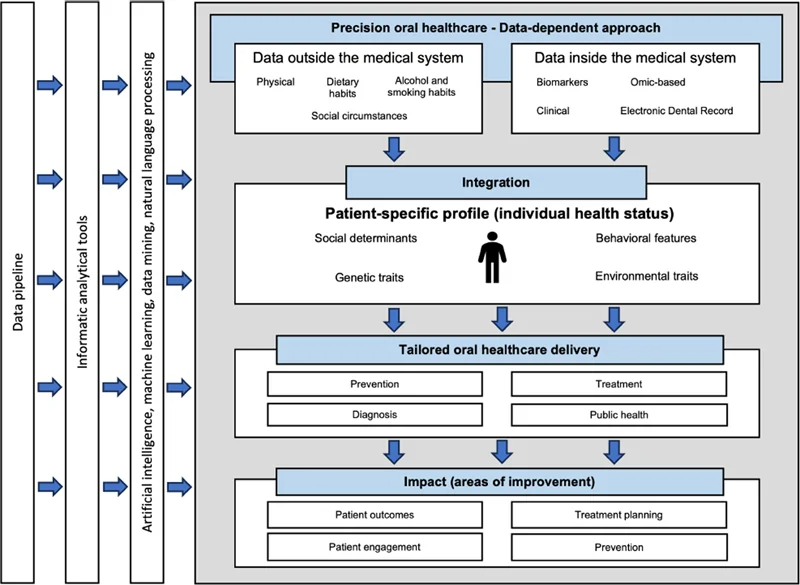
Conceptual framework concerning precision oral healthcare.

The World Health Assembly has released resolutions urging member states to adopt policies and legislative mechanisms related to a national eHealth strategy [ 8 ]. The resolutions also call for promoting the use of international and open standards to ensure the interoperability of digital technologies for health, which is an affordable, effective, and easily adaptable solution. Additionally, appropriate legislation and data protection policies should be developed around issues such as data access, sharing, consent, security, privacy, interoperability, and inclusivity [ 9 ]. In this context, eHealth refers to the cost-effective and secure use of information and communications technologies to support health and related fields, including healthcare services, health surveillance, health literature, and health education, knowledge, and research [ 10 11 ]. In 2021, the World Health Organization released the Global Strategy on Digital Health 2020 – 2025 [ 10 ], which outlines four strategic objectives related to 1) Global collaboration and advancing the transfer of knowledge on digital health; 2) Advancing the implementation of national digital health strategies; 3) Strengthening governance for digital health at global, regional, and national levels and; 4) Advocacy for people-centered health systems that are enabled by digital health. A scoping review conducted by Benoit *et al.* [ 12 ] on the current state of dental informatics looked at the progress made over the last 50 years and found most progress was limited to the US, and very little was done towards reusing EHR data for clinical decision support. Some of the challenges and opportunities reported concerning precision dentistry are shown in Table 1 . Recommendations for precision oral healthcare are shown in Table 2 .

**Table 1.**
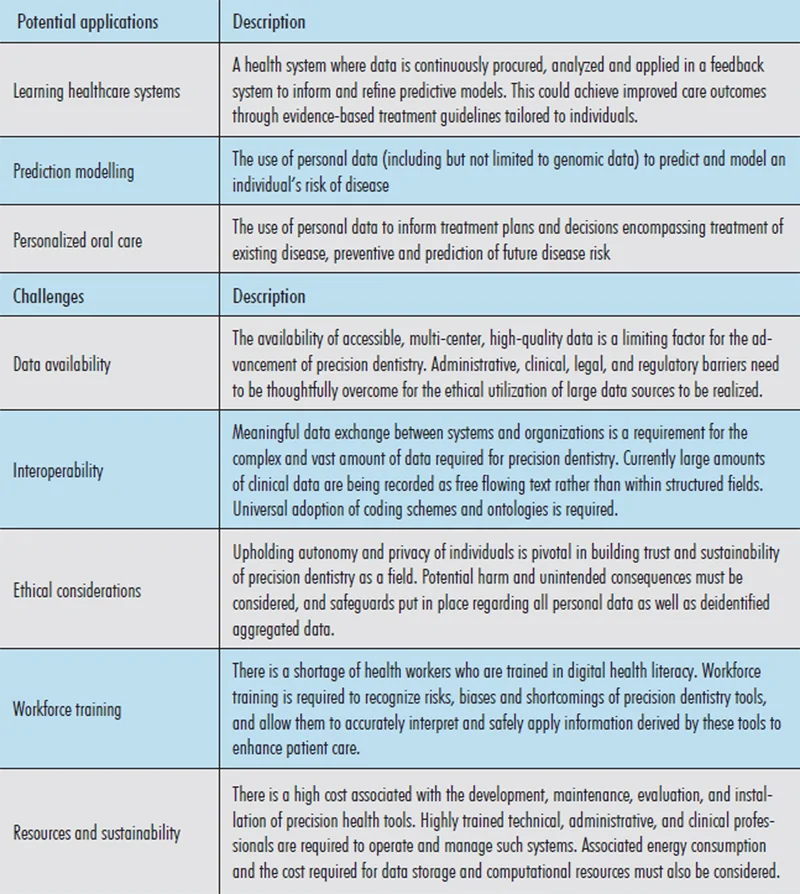
Potential applications and challenges concerning precision dentistry

**Table 2.**
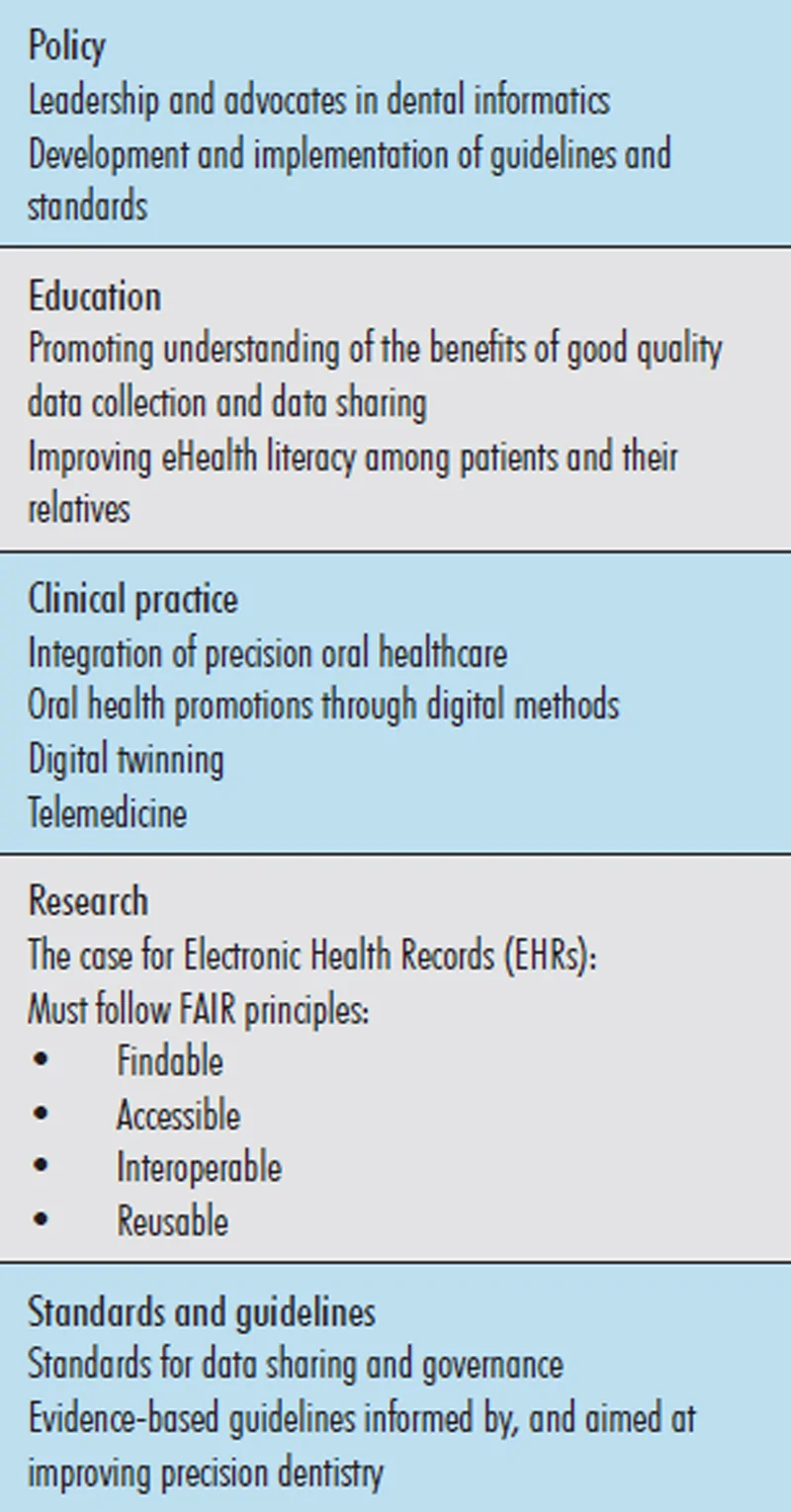
Recommendations for Precision Oral Healthcare

## 2. Potential Applications

### 2.1. Learning Healthcare Systems

Learning healthcare systems create a feedback loop where data from ongoing healthcare is continuously analyzed to improve future care [ 13 ]. A scoping review by Finkelstein *et al.* [ 14 ] explored using big data from these systems to promote precision oral health. They found many applications for such data, including identifying risk factors for periodontal disease, predicting rare adverse events, generating tooth survival curves, data mining for periodontal disease predictors, and evaluating periodontal treatment outcomes. Other analyses of electronic health record (EHR) data focused on risk factors for edentulism, impact of obesity on oral cancer, outcomes of posterior restorations, and outcomes of preventive dentistry visits. They concluded that big data from learning healthcare systems enables diverse analytical applications to support precision and personalized oral healthcare through improved risk assessment, treatment planning, and outcome prediction for each patient. As more data is aggregated over time, the feedback loop will continue refining predictive models and evidence-based treatment guidelines tailored to individuals [ 14 ]. A notable example of an existing repository is the multi-institutional dental data repository, BigMouth, which is available for research and advancing evidence-based dentistry. BigMouth is an excellent model for aggregated dental data repositories that can enable precision oral healthcare through data sharing and analysis [ 15 ].

### 2.2. Prediction Modeling

Prediction modeling plays a crucial role in enabling precision oral health. Such models have been developed to predict outcomes like caries risk, periodontal disease progression, caries lesion advancement, and tooth loss. For caries, tools like the Cariogram incorporate multiple factors to estimate an individual's future caries risk. The full and simplified Cariogram has demonstrated reliability in predicting caries increment clinically and community wide. The simplified Cariogram may be preferred for more straightforward implementation [ 16 17 ]. For periodontal diseases, the Periodontal Risk Assessment (PRA) and Periodontal Risk Calculator (PRC) can predict disease progression and tooth loss across diverse populations after treatment [ 18 ]. However, some models, like those predicting tooth loss during periodontal maintenance therapy, can have low accuracy and generalizability, as was found in a systematic review conducted by Schwendicke *et al.* [ 19 ]. Other tools aim to estimate risks for overall oral health. For example, the Health Improvement in Dental Practice (HIDEP) model uses predefined risk groups to guide treatment and prevention planning. The PRC focused on periodontal risks and was later expanded into the more comprehensive PreViser tool [ 18 ]. Prediction modeling shows promise for enabling precision dentistry when carefully validated. Ongoing development and assessment of risk models are essential to ensure accuracy and utility in clinical practice. Integrating these personalized risk assessments into treatment planning can better target preventive interventions and improve oral health outcomes. Further research is still needed on utilizing risk assessment to improve patient management.

### 2.3 Artificial Intelligence

Artificial Intelligence (AI)specifically deep learning approaches in computer vision, has gained traction in dentistry for automated image analysis. Deep learning algorithms can extract images' features like color, texture, edges, shapes, and structures. For example, deep learning has been used to detect and classify anatomical landmarks, including individual teeth, on various radiographs like cephalometrics, panoramics, and 3D CBCT (cone beam computed tomography) scans [ 20 ]. Automated landmark detection can aid treatment planning and analysis, requiring detailed tooth segmentation. These AI systems can also identify pathological conditions on dental images. Studies have demonstrated the accurate detection of apical lesions, periodontal bone loss, and caries lesions on radiographs comparable or superior to human dentists. AI can also identify restorations and screen for issues like osteoporosis on X-rays [ 20 ]. Beyond diagnostics, some research has focused on using AI algorithms to predict growth patterns and skeletal relationships from dental radiographs, which can support orthodontic treatment planning and evaluation. While AI and deep learning applications in dentistry are still evolving, the future is promising. Automated dental image analysis can improve the efficiency, consistency, and accuracy of diagnostic, prognostic, and treatment planning processes. With further development, AI could become a standard tool used in everyday precision dentistry, enhancing clinical decision-making and outcomes through personalized interpretation of each patient's unique imaging data.

### 2.4. Personalized Oral Healthcare

Traditional one-size-fits-all health strategies are being replaced by precision approaches tailored to each person's unique genetic, biological, and social characteristics [ 6 ]. Extending precision medicine principles to oral health enables more efficient integration of oral and systemic care [ 21 ]. Dentistry is undergoing a transformation driven by advancing scientific understanding of oral diseases and technological innovations in diagnostics and therapy. There is a growing need to apply a range of evidence-based concepts in a highly individualized manner to increase effectiveness and efficiency. Treatment strategies should be tailored not only to the patient's age and risk profile but also to the specifics of each tooth, site, and lesion [ 22 ]. For example, while dental caries were previously assumed only to affect younger populations, we now know older adults experience different carious lesions, such as root caries.

Risk assessment is also becoming a critical component of care, as management should be adapted to both overall patient risk and the particulars of each affected tooth surface and lesion. Factors like lesion depth, surface, status, and activity guide optimal treatment selection from the many options available. Current approaches often stratify patients by age, risk, or lesions based on a few key parameters that are readily accessible to dentists. However, expanding data sources like genomic, microbiomic, proteomic, and metabolomic profiles from each individual, along with insurance and health records, will enable more comprehensive precision care. With improved computing power to analyze large datasets, dentists can understand and modify risk early for better long-term oral health outcomes rather than just managing established diseases [ 22 ].

Patient-specific modeling (PSM) involves the development of computational models of human pathophysiology tailored to patient-specific data. PSM shows promise for improving diagnosis, optimizing treatments by predicting outcomes, aiding in surgical interventions, and developing training tools [ 23 ]. However, while PSM research has gained popularity, with many research groups worldwide recognizing its potential, it is not yet standard clinical practice. Widespread adoption will require large-scale validation of PSM's predictive capabilities on real patient populations and demonstration of tangible clinical utility [ 24 ]. Though initial results are promising, thoroughly evaluating the accuracy and value of individualized models across diverse settings and scenarios is still needed. Once proven, PSM could become integral to precision medicine - developing virtual models of each patient to simulate interventions and enhance care planning. Genomics will also play an increasingly important role in preventing and treating periodontitis [ 25 ]. Similarly, personalized dentistry may also have applications in orthodontics, as the etiology of malocclusions is highly dependent on both genetic and environmental causes [ 26 ].

## 3. Challenges and Ethical Considerations

Precision dentistry is an emerging field at the intersection of dentistry and personalized medicine. In the pursuit of high-quality, personalized oral healthcare service delivery, several formidable challenges emerge, prominently concerning data availability, interoperability, ethical considerations, and the need for a skilled workforce, resources, and sustainability. Table 1 summarizes the current challenges encountered in the field of precision dentistry.

### 3.1. Data availability

The success of precision dentistry hinges on data availability and the quality of obtainable data while also navigating the regulatory landscape. Advancement of the field relies on the acquisition and analysis of vast amounts of data, including genetic information, oral microbiome profiles, and clinical records. Data availability can be a stumbling block, as not all patients can obtain comprehensive dental records or access to standardized testing. Data quality can vary widely, with inconsistencies in data collection methods and potential biases. Current risk prediction models rely heavily on clinically determined phenotypes of a patient such as a clinician's observation of a tooth or a periodontal pocket and are mostly updated manually at each visit to the dental practice. Information on risk indicators rely on self-reported history such as dietary habits and oral home care routines (ADA, 2009). Schwendicke & Krois [ 26 ] argue that current oral and dental research models have been built on parameters that experts determine as important and only these parameters are captured at single time points (at dental visits) without reflecting on changes in the status of comprehensive risk indicators over time. Ensuring that the data used in precision oral health care is accurate, representative, and up-to-date is crucial to its advancement and application in clinical practice.

Local and national legislation on data security and privacy demands meticulous consideration for advancements in preventive healthcare. There is a resounding call for evidence-based oral healthcare guidelines to advance precision oral healthcare delivery. These aspirations, however, grapple with critical issues like the scarcity of up-to-date evidence, few available integrative analytical data sets, and the slow transition of research findings into standard care practices [ 14 ]. Opt-out data policies adopted by organizations such as the National Health Service (NHS) increase data-availability compared to opt-in systems. This means that patient data is, by default, available for research and service planning unless patients or people acting on their behalf opt out of sharing all or parts of their personal information [ 27 ]. This extends only to services provided through the NHS and does not encompass services provided by the private sector which account for at least 40% of dental practices in the United Kingdom (inclusive of fully private and mostly private practices) [ 28 ]. The NHS report that 18.1 million adults were seen by a NHS dentist in the 24-months leading up to 30 June 2023. This is approximately 36.9 percent of the adult population. However, it is difficult to see the full picture of how much of the population accessed dental care services, without integrated data from private practices. Current estimates rely on patient surveys such as the 2020 GP Patient Survey, which produce cross-sectional, self-reported data with high drop-out rates, introducing opportunities for bias [ 29 ]. With integrated data from private practices, it will be a lot easier to gauge an accurate picture of the dental health of the population, access to care for evaluation, and planning of services.

The availability of multi-center data and the potential for data triangulation across diverse sources, encompassing imagery, text-based data, omics data, behavioral patterns, and environmental data, stands as a beacon for the future of oral healthcare [ 26 ]. In this landscape, meticulous attention to both the legal framework and the data quality becomes paramount as precision dentistry evolves to meet the growing challenges and opportunities of our data-rich, technologically advanced healthcare environment. As data-driven technologies permeate healthcare, incorporating AI and data from Internet of Things (IoT) devices such as wearables, ingestibles, implantables, and electronic health records (EHRs), new data sources proliferate. Patients armed with wearables and willing to share data from various aspects of their lives can actively contribute to their personalized healthcare journey. The expanding data sources mean that healthcare decisions do not solely rely on data collection in clinical settings, but patients can choose to donate their data, ranging from behavioral diaries, healthcare applications, tooth brushing motion trackers, etc., to become partners and active shapers of their healthcare outcomes. The data obtained from these novel data sources can be automatically categorized and analyzed using natural language processing, promising more precise and tailored treatments appropriate for the individual [ 26 ].

### 3.2. Interoperability

The absence of widely implemented standards for data exchange and re-usage is a significant hurdle, leading to a need for proper indexing in oral health data. There is limited semantic interoperability, which is required to provide integration and meaningful use of healthcare data between organizations, systems, clinicians, and patients [ 30 ]. Underutilization of existing systems like SNOMED CT (Systematized Nomenclature of Medicine Clinical Terms) overlooks a way for the easy exchange of data. This results in a vast amount of digitized medical data remaining in silos, despite its theoretical accessibility [ 26 ]. SNOMED CT is one of the most robust ontologies; it is a coding scheme for identifying terms and offers a multi-hierarchical structure for relating concepts. SNOMED CT has seen increasing implementation in practical healthcare settings since the early 2010s, with more than 50 countries now widely encouraging its use. However, it is noted that there is a gap in the literature demonstrating the impact and efficacy of SNOMED CT usage on clinical outcomes. This emphasizes the need to investigate whether its application genuinely enhances patient care [ 31 ]. Moreover, while SNOMED CT is a valuable resource, some studies found that it lacked the necessary definitional expressions to describe clinical phenotypes accurately [ 31 ]. This limitation may stem from factors such as the type of text from which concepts are extracted and the use of modifiers. Extensive efforts have been made to extend SNOMED CT's coverage, including the use of post-coordinations and the development of subsets and reference sets. However, localized attempts to improve content coverage should not compromise the quality and scalability of this extensive ontology. Addressing the challenge of interoperability in precision oral health care requires a more comprehensive exploration of standardized terminologies like SNOMED CT and ongoing efforts to bridge the semantic gaps in clinical data exchange.

### 3.3. Ethical Considerations

Precision oral health care aims to tailor therapy to an individual's biological, social, and behavioral characteristics. This approach necessitates comparing an individual's data with that of large, diverse populations to identify subgroups likely to respond differently to different therapeutic modalities. Demographic and socioeconomic data such as ethnicity, housing, education, lifestyle, are integral layers to the complexity of data needed for tailored therapy [ 32 ]. Biological data such as genomic, microbiomic, and proteomic data are also required to predict which therapy may be most efficacious, efficient and safe for an individual [ 26 ]. However, the ethical landscape grows more intricate with the investment in projects like the Human Genome Project (HGP), which spurred legal and ethical considerations. Upholding patient or participant privacy becomes paramount when utilizing a person's genetic data for research or therapeutic applications, demanding safeguards against genetic discrimination abuse in various domains [ 33 ]. The unintended consequences of precision oral health, particularly the potential exacerbation of oral health disparities, cannot be ignored. The uptake of novel oral health services may be more challenging for groups with limited oral health literacy and inadequate insurance coverage. The detailed genomic data collected could also lead to discrimination in insurance coverage and employment, as individuals at higher risk for chronic medical conditions could be identified and denied affordable insurance coverage [ 32 34 35 ]. The intersection between government-held and individual-generated data from wearable devices adds another layer of ethical complexity. The public trusts government agencies over private businesses in safeguarding their information. Patients may consent to access data collected through wearable devices by a healthcare provider working for government agencies without realizing the extent to which their data may be shared with private businesses for quality control [ 36 ]. This necessitates transparent communication and privacy safeguards to ensure patient autonomy is upheld. In the journey towards precision dentistry, navigating these ethical considerations is imperative to safeguard patient privacy, promote equity, and maintain trust in the healthcare system while reaping the benefits of personalized healthcare.

### 3.4. Workforce Training

The challenges confronting precision dentistry include the dearth of a skilled and well-prepared workforce. Enhancing digital health literacy among healthcare workers is crucial. Many advanced prediction models, particularly those capable of leveraging vast and intricate datasets, operate as “black boxes.” How outcomes are derived remains opaque, making it challenging to evaluate them for biases and reasoning. The datasets utilized for predictive modeling should also undergo rigorous scrutiny to identify potential biases, such as sample selection bias, which can significantly impact the models' generalizability and fairness. It is essential to recognize that bias can also emerge from the users of these prediction models. Factors like confirmation and automation bias, where users unquestioningly follow automated suggestions without due attention, are particularly concerning, especially when dealing with multiple tasks simultaneously. This underscores the need for education and awareness, ensuring that users are equipped with the knowledge to interpret probabilistic input information from these models [ 26 ]. Addressing these challenges requires a concerted effort to bridge the workforce gap, foster digital health literacy, and improve the transparency and fairness of precision oral health care.

### 3.5. Resources and Sustainability

Resource scarcity and sustainability pose significant challenges in the realm of precision oral health care. The potential of finite-element (FE) models to consider individualistic parameters for treatment planning in dentistry has been recognized for over three decades. Imaging technologies such as cone-beam computer tomography (CBCT) further enable clinicians and researchers to gather an array of patient-specific characteristics with the accuracy required for patient-specific modeling (PSM). This produces digital twins that can simulate the biomechanics of different therapeutic procedures for a given individual and allow the treatment to be adapted accordingly. Practical application of technologies like this has been hindered, however, by the need for powerful computer hardware, specialized software, and operator expertise [ 37 ]. However, recent advancements in artificial intelligence (AI) have streamlined the process, automating various aspects of PSM, including anatomical segmentations and computer-aided design (CAD). This has not only reduced the time and expertise required but has also made these digital tools more accessible to clinicians and researchers [ 37 ]. Predictive modeling and precision oral health care necessitate extensive data storage, transmission, and computation. The required technological resources have shown remarkable growth rates over the recent years. It is vital to acknowledge the associated costs and energy consumption for training and running these models, highlighting the need for a critical evaluation of resource utilization and sustainability in the field [ 26 37 ].

## 4. Conclusions

Personalized or precision healthcare reflects the expectation that advances in genomic, imaging, and other domains will extend our risk assessment, diagnostic, and prognostic capabilities and enable more effective prevention and therapeutic options than inequity. With increasing awareness of the importance of dental informatics, as well as the development of safe, ethical, and effective data models, the vision of precision prevention in dentistry can be realized.
